# Modeling HIV persistence and cure studies

**DOI:** 10.1097/COH.0000000000000490

**Published:** 2018-05-08

**Authors:** Alison L. Hill

**Affiliations:** Program for Evolutionary Dynamics, Harvard University, Cambridge, Massachusetts, USA

**Keywords:** HIV, latency, mathematical modeling, viral dynamics

## Abstract

**Purpose of review:**

To provide a summary of the contributions of mathematical modeling to understanding of HIV persistence during antiretroviral therapy.

**Recent findings:**

Although HIV persistence during therapy could be caused by continual viral replication or slow-decaying latent infection, most evidence points toward the latter mechanism. The latent reservoir is maintained by a balance of cell death, proliferation, and reactivation, and new methods to estimate the relative contributions of these rates use a wide range of experimental data. This has led to new quantitative predictions about the potential benefit of therapies such as latency-reversing agents or antiproliferative drugs.

**Summary:**

Results of these mathematical modeling studies can be used to design and interpret future trials of new therapies targeting HIV persistence.

## INTRODUCTION

HIV infection cannot be cured by antiretroviral therapy (ART) alone, which hinders efforts to control the epidemic worldwide. To accelerate progress toward a cure, we must understand the mechanisms responsible for maintaining the virus despite therapy, and use this understanding to make informed decisions about which types of new therapies could effectively perturb or purge this reservoir [1]. One of the lesser known tools to address these challenges is mathematical models, which are sets of equations or rules describing how populations – of proteins, viruses, cells, people, so on – interact and change over time [2]. Models have a long history of use to describe the spread of infectious diseases in populations [3], and have been adopted to explain the ‘viral dynamics’ of HIV within infected individuals since the virus was first recognized [4]. 

**Box 1:**
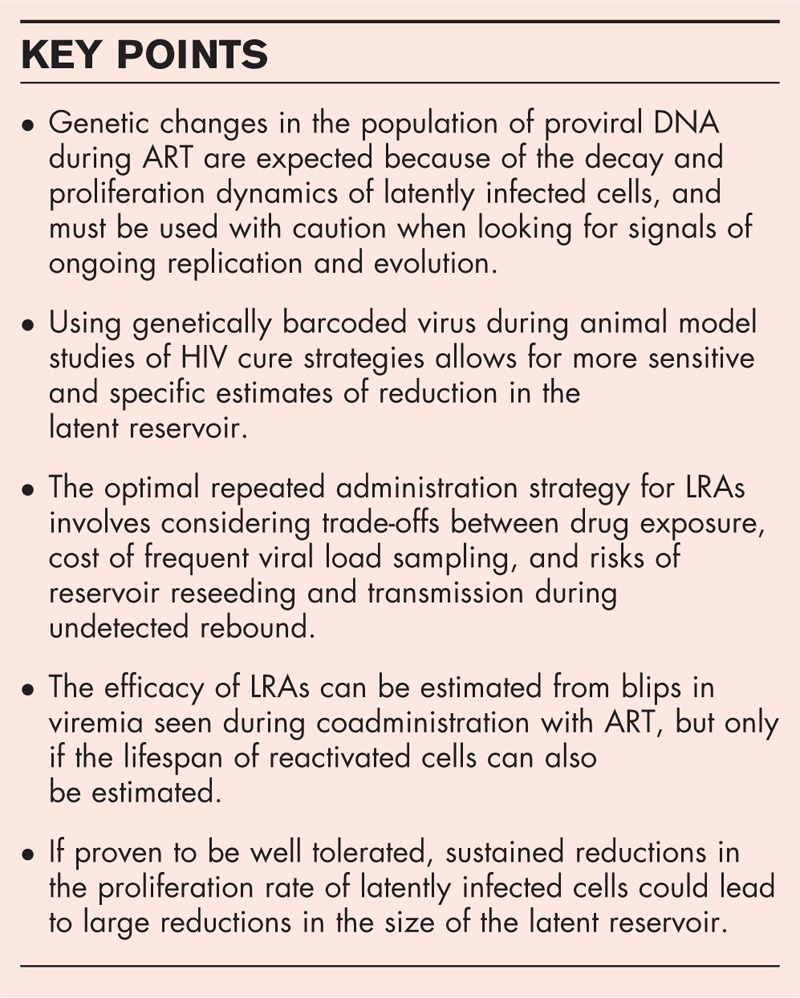
no caption available

Related to HIV persistence, previous models have explained the multiphasic decay of viral load during ART [5], the initial seeding of the latent reservoir during acute infection [6] and the limited inflow during treatment [7], the impact of interventions to reduce the latent reservoir [8–10], and the potential role of ongoing replication [11^▪^,^12^] (reviewed in [13]). Overall, mathematical models offer a formal way to integrate previous knowledge and make predictions about how a system's behavior might change under different interventions.

In this paper, I will review recent work over the past 2 years applying mathematical models to understand issues related to HIV latency and persistence. This work centers around three main questions:

(1) What is the mechanism of viral persistence despite ART? In particular, a major debate has centered around the role of ongoing viral replication because of suboptimal therapy efficacy, versus the persistence of latently infected cells. Mathematical models and population genetic analysis have been used – with increasing frequency over the past few years – to support or refute these hypotheses. As most studies support the sole role of latency, much of the other work that will be reviewed presupposes latency as the main barrier to cure.
(2) How often do latently infected cells reactivate? One of the major determinants of the timing of viral rebound, and the expectation for how it should increase and eventually cease if new interventions can purge the latent reservoir, is the rate at which latently infected cells naturally reactivate and restart producing virions. This cellular event is not directly observable *in vivo*, and so various indirect ways of inferring this quantity have been proposed, with heated debate over their relative merits and pitfalls. Recently, a new experimental system based on barcoded virus has been devised that allows for more accurate way of probing the rate of latent cell reactivation and how it depends on reservoir size.
(3) What is the predicted effect of therapies aimed at disrupting viral persistence? Over the past decade, research has been underway to develop new pharmaceutics that prevent or delay viral rebound when ART is stopped. So far, these attempts have been unsuccessful. Mathematical models can be used to predict the conditions under which therapies have the most promise, and help interpret viral kinetics during drug administration. Recent work has provided important insights into the potential of three new classes of drugs: latency-reversing agents (LRAs), antiproliferative therapy, and pro-trafficking drugs.

## MECHANISMS OF PERSISTENCE OF HIV DURING ANTIRETROVIRAL THERAPY

Individuals who adhere to combination ART for decades still experience low-level residual viremia and rapid rebound of infection upon cessation of therapy. As this fact emerged nearly 20 years ago, two competing explanations have emerged: one, that ART is unable to completely suppress viral replication throughout the body, and two, that latently infected cells persist for extremely long times. The evidence for and against each stance has been reviewed many times in the past, including recently by van Zyl *et al.*[14], with the conclusion that most of the evidence supports the role of latency.

A recent study by Lorenzo-Redondo *et al.*[15] has challenged this view, by finding signals of continual evolution in viral sequences sampled longitudinally during ART, which would indicate that viral replication (accompanied by mutation and selection) continues. Although previous studies have looked for and failed to find such signals, Lorenzo-Redondo *et al.*'s [15] approach was unique in including samples from both peripheral blood and lymph tissue, in having multiple samples closely spaced near the time ART was started (0, 3, and 6 months after), and in using newer deep sequencing approaches to capture genetic variation.

Since its publication, multiple independent groups have suggested that the signals of evolution seen by Lorenzo-Redondo *et al.* are more likely to be artifacts of the known dynamics of the provirus-carrying cells themselves, and not related to viral spreading and mutation. The idea is that during untreated infection, virus evolves and diverges rapidly, infecting both short-lived and long-lived cells. Before and during the first few months of ART, shorter-lived infected cells dominate the HIV DNA pool, and as these cells must have been recently infected, they carry the most ‘evolved’ virus population. Longer-lived infected cells, which will be the only ones left during long-term ART, can carry virus generated at any point during untreated infection, including very ancestral strains. As short-lived cells decay to reveal long-lived cells, the provirus population could appear to diversify and diverge over time.

Rosenbloom *et al.*[16] and Brodin *et al.*[17^▪▪^] used simulations of pretreatment viral evolution, seeding of the latent reservoir, fully-effective ART, and longitudinal sampling to show that this mechanism could indeed give false signals of evolution when sequences during ART are compared with those present at the time of ART initiation. These signals can occur with realistic evolutionary dynamics before treatment, with substitution rates that are informed or taken directly from observed pre-ART sequences. Kearney *et al.*[18], Van Zyl *et al.*[19^▪▪^], and Brodin *et al.*[17^▪▪^] have used data generated from their own studies of individuals sampled after up to 10 years on ART – when only long-lived cells remain – to look for evolution, and have seen either no significant genetic divergence or instead ‘reverse evolution’ toward more ancestral viral strains that were sampled during early infection. In individuals who have incomplete adherence/suppression on ART, forward evolution is clearly seen [19^▪▪^]. Together, these recent studies suggest that the evidence for ongoing replication as the dominant mechanism of viral persistence remains limited. An update to this view will require studies that sample sequences longitudinally both pre-ART and greater than 1 year post-ART, and ideally focus on replication-competent provirus and other tissues where drug sanctuaries are proposed to exist.

Even if latency is the dominant mechanism for viral persistence during ART, the question still remains open as to how these cells persist for decades during therapy. The intrinsically long life-span of resting memory CD4^+^ T cells is one possibility, however, recent studies that have examined the clonal composition of the latent reservoir using viral integration sites or near-full length viral sequences (e.g., [20,21], reviewed in [22]) have found multiple identical infected cells, suggesting they must have descended from a single infected cell by the process of cell division. Although these studies *qualitatively* showed that proliferation of latent cells (without reactivating viral production) must be important for persistence, they were unable to quantify its importance, as many latent cells observed to be ‘unique’ may actually be part of clones that simply were not sampled. To address this issue, a recent study by Reeves *et al.*[23] used statistical models taken from ecology to extrapolate the true total-body clonal composition of the latent reservoir from the sampled composition. Under a wide range of assumptions, they found that at least 99.9% of cells in the reservoir were likely descended from proliferation, as opposed to infection, which suggests that if some sort of reservoir-specific antiproliferative therapy were possible, it may lead to large reductions in the latent pool (caveats discussed in last section of study).

## METHODS TO ESTIMATE THE RATE OF REACTIVATION FROM LATENCY

The time to viral rebound when ART is stopped, and how this time will increase as reservoir size decreases, depends on the rate at which latent cells reactivate (and subsequently cause a chain of infection that eventually leads to detectable viremia) [9,10]. This rate has proven difficult to measure directly. The distribution of time to rebound in a cohort can only be used to infer this rate if assumptions are made about inter-patient heterogeneity in reservoir sizes, activation rates, and viral growth rates, and about the establishment probabilities of reactivating lineages [8,9,24,25]. An equally important parameter is the reduction in latent cell reactivation rate by an investigative therapy, but this is difficult to measure as the frequency of latent cells in a limited blood sample is often zero. It can be estimated by fitting the kinetics of viral rebound to mathematical models – a strategy that has been used to understand the reservoir-reducing effects of toll-like receptor 7 (TLR7)-agonists in simian immunodeficiency virus (SIV)-infected macaque [26,27^▪^] – but these inferences are often imprecise and qualitative because of the difficulty of separating out effects of antiviral immune responses and the time for ART to wash out.

One striking observation is that when rebound occurs rapidly from large reservoir sizes, it often contains genetically diverse virus [28], whereas rebound that occurs from undetectable reservoir sizes after long delays is composed of clonal viral populations [29,30]. This suggests that genetic sequencing of rebounding virus could be used to estimate the reservoir size and reduction during therapy, but this strategy is expensive (requires near full-length single-genome analysis of a large population of viruses) and often inconclusive if viral diversity was limited before ART. Inspired by this idea, a combined experimental-modeling team recently developed a strategy of infecting with a swarm of barcoded viruses during animal studies [31^▪▪^], which ensures genetic diversity that can be easily measured with bulk next-generation sequencing.

As a proof-of-concept, the authors compared the number of lineages contributing to rebound when ART was given for only a few months and virus was incompletely suppressed (87–136 clones seen 7 days after ART stop), compared with when ART was started extremely early and continued for around a year (2–6 clones at ∼2 weeks after ART stop). They used the same data to estimate the frequency of latent cell reactivation between the two ART regimes, suggesting ∼20 cells per day reactivating in the first case compared with 1 cell every 2 days in the second case. Although these results cannot be used to estimate reservoir reduction unless one is certain viral replication has been completely suppressed by ART, they suggest that future cure studies in nonhuman primate studies [26,27^▪^,^32^,^33^] would be much easier to interpret if they simply adapted their methods to use barcoded virus stocks.

Subsequent work by some of the same individuals suggest other applications of this system. For example, Pinkevych *et al.*[34] used the kinetics of clones during rebound to estimate the amount of plasma viremia caused by a single reactivating cell, in both SIV (using barcoded virus) and HIV (using natural genetic variation), inferring between 0.1–1 RNA copy/ml. Although this estimate is highly sensitive to a number of modeling assumptions that are difficult to verify, it may be useful for future models of viral rebound. In a recent abstract, Swanstrom *et al.*[35] examined the impact of massive CD4^+^ T cells-depletion administered during long-term suppressive ART. By the time ART was interrupted, CD4^+^ T cell levels had returned to approximately half baseline values. Overall, no significant differences were observed in the time to rebound or the number of rebounding clones between CD4^+^ T cells-depleted and control groups, suggesting that reservoir size, and some level of diversity, can recover from depletion. Interestingly, the animal with the longest delay to rebound (13 weeks) and the one who controlled to very low viral loads after high-peak viremia had only a single clone observed during early rebound.

## POTENTIAL BENEFIT OF THERAPIES TARGETING PERSISTENT HIV INFECTION

Many strategies are currently under investigation to combat HIV persistence, including LRAs such as histone deacetylase (HDAC) inhibitors and protein kinase C agonists, immunotherapies such as TLR7-agonists, monoclonal antibodies, checkpoint blockade inhibitors, and chimeric-antigen-receptor T cells, gene therapies to render target cells resistant to HIV infection or excise integrated provirus, and agents to permanently silence latent infection (reviewed in [1]). However, none of these approaches has so far led to dramatic clinical benefits, and work is needed to better define what strategies are most promising to pursue and what parameters should be optimized to make success most likely.

In the past 2 years, several comprehensive modeling studies have provided important insight into the development of some of these new therapies. Petravic *et al.*[36^▪▪^] and Cromer *et al.*[37] focused on LRAs, which aim to reactivate latently infected cells while a patient is on ART, with the hope that these cells will eventually die and reduce the reservoir. Uncertainties about LRAs include the relationship between the particular amount by which reactivation is increased during therapy, the length of time it is given, and the expected decrease in the latent pool. Petravic *et al.*[36^▪▪^] developed a mathematical model of latent cell dynamics to explain how these variables interact. They showed that depending on the relative magnitude of the rates of latent cell division, death, and reactivation, an LRA that, for example, caused a three-fold increase in reactivation could either cause a less than, greater than, or equal to three-fold increase in reservoir decay rate, making predictions of therapy efficacy impossible until these rates are estimated (which the authors did not attempt to do in this study). Another challenge with LRA therapy is that it is generally impossible to directly measure the impact it had on reducing the latent reservoir, as latently infected cells comprise such a small frequency of all lymphocytes and any blood draw contains small quantities of them. It has been suggested that the increase in plasma viremia or cell-associated RNA seen during LRA administration can be used to back out increase in reactivation rate [38], and hence the expected reduction in the total latent pool. Petravic *et al.*[36^▪▪^] also explored the parameters needed to do this calculation correctly, and highlighted the critical need to know the lifespan of LRA-reactivated cells (which likely differs from naturally reactivated cells). Considering recent clinical trials of HDAC inhibitors panobinostat [39] and romidepsin [40], the authors use their models to explain that while both drugs caused similar increases in HIV RNA in cells, romidepsin was likely much more effective, because the cells it reactivates die quickly. Neither drug was likely efficacious enough to be expected to lead to large decreases in the reservoir, agreeing with observations.

The best-case scenario for LRA therapy is that the reservoir will be reduced to such a small number of cells that the probability that any of them reactivate, escape stochastic extinction, and cause viral rebound during a patient's remaining lifetime is negligible. However, current LRAs are nowhere near this goal and even risky stem cell transplants have failed to prevent rebound for more than a year [29]. For now, it is more likely that the reservoir will be partially reduced, and that patients will have to be closely followed until rebound occurs, at which point ART can be restarted, and perhaps repeated cycles of LRA therapy and treatment interruption conducted.

Cromer *et al.*[37] used a model of rebound their group had previously developed [11^▪^] to examine this strategy, and ask which timing of LRA administration minimized drug exposure: either large in initial LRA dose leading to dramatic reduction in reservoir size, or more moderate initial dose and reduction requiring followed by some repeat dosing. The found that above some dose size (reservoir reduction), there was no benefit to larger initial reservoir reductions. The model did not factor in costs of repeated treatment, such as the need for frequent viral load sampling and the risks of transmission or development of drug resistance, and did not have the possibility of achieving cure without eliminating every single latent cell. The authors also examined how the risk of eventual rebound decreases as the time of remission increases and how (in)frequent viral load sampling can lead to reservoir reseeding during viral rebound (similar to [41] for a different model), and additionally looked at how undetected rebound could contribute to transmission. These different components of their work could be combined in the future to design LRA-administration strategies that weighted the trade-offs between drug exposure and risks associated with uncontrolled rebound.

Given the recent realization that the reservoir is likely maintained by proliferation of latently infected cells [22], therapy that slows the division rate of resting memory CD4^+^ T cells may help reduce and possibly clear the reservoir. Reeves *et al.*[42^▪▪^] constructed a model of reservoir dynamics to examine antiproliferative therapy, and compare it to LRAs. Similar to Petravic *et al.*[36^▪▪^], their model includes latent cell death, division, reactivation, but also allows some low-level subcritical viral replication. They show that modest, sustained increases in latent cell reactivation or decrease in proliferation can lead to large reductions in the latent reservoir after a few years. However, antiproliferative therapy needs much less efficacy to achieve the same reduction, compared with LRAs. For example, they predict that an approximately four-fold decrease in proliferation rate would lead to a 100-fold reduction in reservoir size after a year of therapy. These results depend critically on the authors estimates of model parameters. They reasonably assume that latently infected cells turnover at the same rate as the T cells subsets they infect, which could be wrong if infection alters cell division rates, or, if more or less proliferative cells are preferentially infected. The rate of latent cell reactivation is estimated from the timing of viral rebound, and latent cell death rate is estimated from the overall decay rate of the reservoir on ART. They additionally assume that a hypothetical antiproliferative therapy does not lead to overall CD4^+^ T cells decline, or cause other alterations to CD4^+^ T cells homeostasis, which could trigger compensatory mechanisms that may negate the antiproliferative effect. Although their results suggest a promising new therapeutic strategy, they point out the difficulty in predicting whether such decreases would lead to long-term antiretroviral-free remission or cure, as different modeling studies have arrived at different threshold reservoir sizes required for these outcomes [10,11^▪^].

As discussed above, there is some suggestion that HIV replication may continue despite ART, especially in ‘sanctuary’ sites, anatomical regions where drug penetration may be low [43]. Fryer *et al.*[44^▪^] propose a strategy to reduce viral replication – ideally to subcritical levels – despite the presence of such sanctuaries, without having to improve the distribution of ART. Instead, they suggest that therapies which increase the migration rate of T cells between the peripheral blood and sanctuary tissues could eliminate ongoing replication. They backup their results with a simple two-compartment viral dynamics model, which shows there is a threshold trafficking level above which ongoing replication despite ART becomes unsustainable and infection levels quickly trend to zero. Intuitively, this occurs when infected T cells cannot remain in the drug sanctuary long enough to infect at least one other cell before they die or move to a region where ART prevents infection. As estimates of the baseline rate of lymphocyte trafficking are missing, and particular ‘protrafficking’ agents do not yet exist, these results are currently mainly theoretical but suggest a potentially promising new avenue for cure strategies.

## CONCLUSION

A permanent cure for HIV will require a comprehensive understanding of the mechanisms responsible for viral persistence and new therapeutics that target them. Mathematical models are helping with this task, by interpreting complex patterns seen in data and translating biological mechanisms into predictive frameworks.

Although the debate continues over the relative importance of latent infection versus ongoing replication for HIV persistence, recent mathematical models that include realistic dynamics of infected cells suggest that the observed viral genetic changes during ART are consistent with fully effective therapy. In either case, mathematical modelers have suggested new avenues for cure: reduction in the proliferation rate of latently infected cells, or increased trafficking of T cells in and out of drug sanctuaries.

LRAs are the cure strategy in the most advanced stage of development, but predicting their efficacy requires understanding how frequently latent cells reactivate when ART is stopped and how new therapies might reduce this rate. A new experimental system and analysis framework using barcoded virus allows for more direct estimation of these rates. Mathematical models have better delineated the parameters needed for success of these drugs, and suggested unifying interpretations for previous clinical trials.

## Acknowledgements

None.

### Financial support and sponsorship

This work was supported by National Institutes of Health awards DP5OD019851, P01AI131365, P01AI131385 and Bill & Melinda Gates Foundation award OPP1148627.

### Conflicts of interest

There are no conflicts of interest.

## REFERENCES AND RECOMMENDED READING

Papers of particular interest, published within the annual period of review, have been highlighted as:

▪ of special interest
▪▪ of outstanding interest
